# Development and Characterization of Sawdust-Based Ceramic Membranes for Textile Effluent Treatment

**DOI:** 10.3390/membranes15100298

**Published:** 2025-10-01

**Authors:** Ana Vitória Santos Marques, Antusia dos Santos Barbosa, Larissa Fernandes Maia, Meiry Gláucia Freire Rodrigues, Tellys Lins Almeida Barbosa, Carlos Bruno Barreto Luna

**Affiliations:** 1 Development Laboratory of New Materials, Academic Unit of Chemical Engineering, Center for Science and Technology, Federal University of Campina Grande, Av. Aprígio Veloso, 882—Bodocongó, Campina Grande 58429-970, PB, Brazil; anavitoria.marques@eq.ufcg.edu.br (A.V.S.M.); antusiasb@hotmail.com (A.d.S.B.); larissa.fernanes@tecnico.ufcg.edu.br (L.F.M.); meiry.freire@eq.ufcg.edu.br (M.G.F.R.); 2 Instituto SENAI de Inovação em Energias Renováveis, Av. Capitão-Mor Gouveia, 2770—Lagoa Nova, Natal 59063-400, RN, Brazil; tellysbarbosa@isi-er.com.br; 3 Academic Unit of Materials Engineering, Federal University of Campina Grande, Av. Aprígio Veloso, 882—Bodocongó, Campina Grande 58429-900, PB, Brazil

**Keywords:** low-cost membrane, wood sawdust, adsorption, low energy consumption, textile dyes, wastewater treatment

## Abstract

Membranes were assessed on a bench scale for their performance in methylene blue dye separation. The sawdust, along with Brazilian clay and kaolin, were mixed and compacted by uniaxial pressing and sintered at 650 °C. The membranes were characterized by several techniques, including X-ray diffraction, scanning electron microscopy, porosity, mechanical strength, water uptake, and membrane hydrodynamic permeability. The results demonstrated that the incorporation of sawdust not only altered the pore morphology but also significantly improved water permeation and dye removal efficiency. The ceramic membrane had an average pore diameter of 0.346–0.622 µm and porosities ranging from 40.85 to 42.96%. The membranes were applied to the microfiltration of synthetic effluent containing methylene blue (MB) and, additionally, subjected to investigation of their adsorptive capacity. All membrane variants showed high hydrophilicity (contact angles < 60°) and achieved MB rejection efficiencies higher than 96%, demonstrating their efficiency in treating dye-contaminated effluents. Batch adsorption using ceramic membranes (M0–M3) removed 34.0–41.2% of methylene blue. Adsorption behavior fitted both Langmuir and Freundlich models, indicating mixed mono- and multilayer mechanisms. FTIR confirmed electrostatic interactions, hydrogen bonding, and possible π–π interactions in dye retention.

## 1. Introduction

Water scarcity and contamination are pressing global challenges [1]. In recent years, different types of membranes have been employed for water treatment, including polymeric and ceramic membranes. Polymeric membranes are particularly widespread in large-scale applications because of their low cost [2], flexibility, and high separation efficiency. For instance, nanofiltration polymeric membranes, such as modified polyethersulfone (PES), have shown high efficiency in the removal of heavy metals from industrial wastewater, maintaining stability under different operational conditions [3]. Despite their advantages, polymeric membranes generally exhibit limited thermal and chemical resistance, as well as a higher susceptibility to fouling [4,5]. Consequently, ceramic membranes have emerged as promising alternatives due to their superior robustness, durability, and ability to operate under harsh conditions [6,7,8,9]. Ceramic membranes are inorganic membranes known for their robustness, durability, resistance to chemical and thermal degradation, and effectiveness in contaminant removal [7,8,9]. Methylene blue (MB) is a commonly used cationic dye that can form stable solutions with water under ambient conditions [10].

Currently, a variety of physical, chemical, and biological treatment methods are widely used to manage dye-containing wastewater. However, these methods have some drawbacks, such as increased energy consumption, high costs, and the generation of large amounts of toxic byproducts. Therefore, the membrane separation process offers distinct advantages [11].

In water treatment, ceramic membranes are used in various processes, including microfiltration, ultrafiltration, nanofiltration, or reverse osmosis, depending on the specific application requirements [12]. Membranes are used in a variety of environments, from municipal water treatment to industrial processes such as wastewater treatment, desalination, and food and beverage production [13].

Ceramic membranes offer several characteristics, such as the ability to operate effectively under harsh conditions, making them suitable for treating water with high temperatures or aggressive chemicals [9]. Furthermore, ceramic membranes offer long-term cost benefits due to their robustness, durability, and minimal maintenance requirements compared to alternative membrane materials. They have a longer service life and are less prone to fouling, thus reducing downtime and operating costs [6,14,15]. However, despite their several benefits, ceramic membranes also face challenges such as high production costs and the need for specialized expertise in manufacturing and installation [16]. Research and development efforts continue to increase their efficiency, reduce costs, and expand their applicability to combat water scarcity and contamination worldwide [17].

Combining kaolin and wood sawdust to create low-cost membranes represents an innovative approach to waste-as-resource utilization, particularly for wastewater treatment applications such as textile dye removal [18]. This method aligns well with sustainability goals, repurposing abundant natural materials while simultaneously addressing environmental challenges [19]. Kaolin, a type of clay mineral, can serve as a base material for the membrane, conferring properties such as porosity and adsorption capacity [20,21,22]. On the other hand, sawdust can increase the structural integrity of the membrane and potentially contribute to its filtration properties [23,24].

Wood processing industries have traditionally faced the challenge of managing sawdust, often resorting to disposal methods that contribute to pollution and resource depletion. However, by repurposing sawdust as a valuable resource, companies can implement strategies that not only alleviate environmental contamination but also provide economic benefits. The process of creating these low-cost membranes typically involves mixing kaolin and sawdust with binders and other additives, forming a composite material that can be molded into membrane modules [23,24]. These modules can then be integrated into wastewater treatment systems to remove textile dyes and other contaminants [25]. The use of these membranes offers several advantages, including cost-effectiveness, environmental sustainability, efficient wastewater treatment, and versatility [26].

Membranes can effectively remove textile dyes and other pollutants from wastewater streams, thereby improving water quality and environmental protection [27].

This study evaluates the production of low-cost ceramic membranes using kaolin and quartz through the tape casting technique, a common method in industry. The researchers assessed the influence of variables such as the amount of quartz, the sintering temperature, and the addition of pore-forming agents (graphite or PMMA) on the membrane properties. The results showed that adjustments in the paste composition and sintering temperature improve membrane characteristics, including porosity and water permeability. In particular, the addition of graphite proved effective in increasing porosity and permeability, although it can result in greater surface roughness. The study concludes that it is possible to produce more affordable ceramic membranes from abundant raw materials, extending their applications in separation processes such as microfiltration and contributing to reduced costs and environmental impact [28].

The main objective of this research was to develop low-cost ceramic membranes using Brazilian clay, kaolin, and sawdust, the latter serving as a pore-forming agent, with a focus on the removal of methylene blue dye from synthetic wastewater. The membranes produced were applied in microfiltration tests with synthetic effluent containing methylene blue (MB) and were also investigated for their adsorption capacity.

This study further aimed to emphasize the importance of carefully selecting accessible and abundant raw materials for the formulation of low-cost ceramic membranes. It is worth noting that studies on low-temperature sintering remain limited, with some reported at 700 °C [29]. In this context, the present work provides an innovative contribution by reducing the sintering temperature to 650 °C and assessing its impact on the properties and performance of the membranes.

## 2. Materials and Methods

### 2.1. Preparation of Ceramic Membrane

In this study, the raw materials used were Brazilian clay, kaolin, and sawdust powder, all sieved through an ABNT 100 (Granutest, Bom Retiro, Brazil) mesh sieve. Table 1 shows the proportions of the initial components employed in the preparation of the ceramic mixtures. The selection of these materials was guided by previous studies conducted by the research group [30,31].

After formulating the mixtures, homogenization was performed with the incorporation of 10% moisture. This step was performed in a ball mill (Quimis, Q298-2, Diadema, Brazil) for 1 h at high speed for each composition. The resulting mixture was then placed in a stainless steel mold and pressed in a hydraulic press (BOVENAU, Rio do Sul, Brazil) using the uniaxial dry pressing method [32], applying a pressure of 1 ton for 1 min.

After compaction, disc-shaped membranes were formed. The pressed discs were dried at 100 °C for 24 h in an oven to completely remove residual moisture. Subsequently, they were subjected to sintering in a muffle furnace (Quimis, Q318M, Diadema, Brazil), which represented the final step in the ceramic membrane production process. Sintering was carried out at 650 °C, with a heating rate of 5 °C/min, and maintained for 3 h.

**Figure 1:**
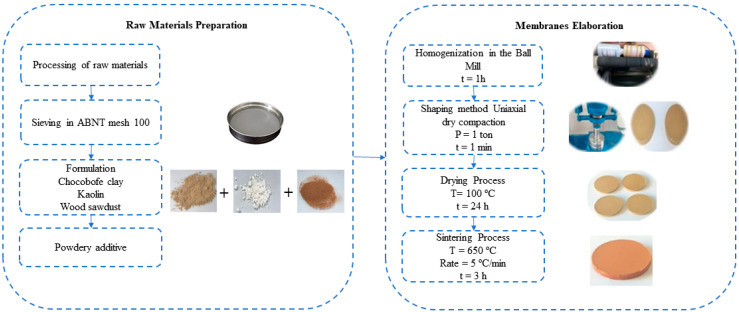
Flowchart illustrating the preparation procedure for the ceramic membrane.

Figure 1 illustrates the manufacturing process of the ceramic membranes.

### 2.2. Characterization

Chemical composition was determined by X-ray fluorescence (XRF) using a (Shimadzu, EDX-720, Kyoto, Japan) spectrometer equipped with an Rh target tube.

The mean particle size of the raw materials was analyzed with a (Bettersize ST Laser, Dandong, China) Particle Size Analyzer.

Fourier Transform Infrared Spectroscopy (FTIR) analyses were performed on a (Bruker, Vertex 70, Billerica, MA, USA) spectrometer in the range of 4000–400 cm^−1^ with 32 scans with a resolution of 4 cm^−1^.

Thermogravimetric (TG) measurements were obtained using a (Shimadzu, DTG-60H, Kyoto, Japan) under a nitrogen atmosphere (100 mL/min), with a heating rate of 10 °C/min, from 40 to 900 °C. Approximately 10 mg of each sample was placed in open alumina crucibles.

X-ray diffraction (XRD) patterns were recorded on a (Shimadzu, XRD 6000, Kyoto, Japan) diffractometer using Cu Kα radiation (40 kV, 30 mA), with a step size of 0.020° and a scan range of 2θ = 2–50°.

The microstructural morphology was examined by scanning electron microscopy (Tescan VEGA, Brno, Czech Republic).

The mechanical strength of the membranes was determined by diametral compression according to ASTM C158, using a universal testing machine (Shimadzu, Kyoto, Japan).

Apparent porosity (*ε*) was measured by the immersion method, based on Archimedes’ principle, following [33]. Tests were performed in triplicate. Porosity was calculated using Equation (1) [31]:

(1) $$\varepsilon =\left(\frac{M_{w}-M_{d}}{M_{w}-M_{i}}\right)\times 100$$

where *M_w_*: wet mass; *M_d_*: dry mass; *M_i_*: immersed mass.

The contact angle was measured with a kinetic goniometer (Alcalitech, AGC 002, Campina Grande, Brazil) with a calibration factor of 0.017. A 20 μL droplet of water was deposited on the sample surface, and the measurement was taken after 15 s at 25 ± 2 °C.

The average pore radius (*r*) of the membranes was estimated using the Guerout–Elford–Ferry Equation (2):

(2) $$r=\sqrt{\frac{(2.9-1.75\varepsilon )\times 8\times \eta \times h}{\varepsilon \times ∆P}\times J}$$

where *ε* is porosity, *η* the viscosity of water at ambient conditions (Pa·s), *h* the membrane thickness (m), Δ*P* the applied pressure (Pa), and *J* the average pure water flux (m^3^·s^−1^·m^−2^).

The hydrodynamic permeability of the membrane (*Lp*) will be determined from the ratio between the water flux (*J*) and the applied transmembrane pressure (Δ*P*) [34]. This approach is expressed by Equation (3):

(3) $$L_{p}=\frac{J}{∆P}$$

where *J* is the permeate water flux (L·m^−2^·h^−1^) and Δ*P* is the transmembrane pressure (bar). This equation allows the direct calculation of the membrane permeability from experimental data obtained in filtration tests.

The water absorption capacity of the membranes was evaluated by measuring the difference between their wet and dry weights. The water uptake was calculated using Equation (4) [35]:

(4) $$WU=\left(\frac{M_{w}-M_{d}}{M_{d}}\right)\times 100$$

where *WU* is the water uptake (%), *M_w_* is the wet mass, and *M_d_* is the dry mass.

### 2.3. Permeation Performance

**Figure 2:**
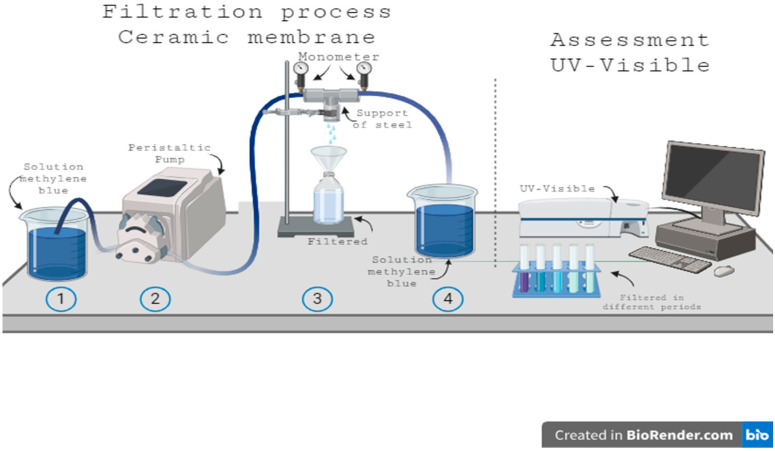
The permeation/separation system was utilized to assess the permeability and selectivity of ceramic membranes.

The performance of the ceramic membrane regarding permeate flux and pure water flux was evaluated in a continuous-flow separation system (Figure 2).

The permeate volume collected after the feed passed through the permeation/separation module was measured every 5 min over 90 min. Transmembrane pressure (ΔP) was monitored using a manometer (bar).

The water flux (*J*) was calculated according to Equation (5) [31]:

(5) $$J=\frac{V}{A\times ∆t}$$

where *J* is the water flux (m^3^·m^−2^·h^−1^), *V* the permeate volume (m^3^), *A* the membrane area (m^2^), and Δ*t* the permeation time (h).

Methylene blue (100 mg·L^−1^) was used to evaluate the rejection coefficient of the ceramic membranes. Filtration experiments were conducted for 90 min at room temperature under a transmembrane pressure of 2 bar.

The rejection rate (*R*%) was calculated according to Equation (6) [31]:

(6) $$R\%=\left(\frac{C_{0}-C}{C_{0}}\right)\times 100$$

where *R*% is the rejection coefficient, *C*_0_ the initial dye concentration (mg·L^−1^), and *C* the final dye concentration (mg·L^−1^).

#### Data Analysis

Flow measurements were obtained at 18 points for each of the four membranes (M0, M1, M2, and M3). Prior to applying the analysis of variance (ANOVA), the assumptions of normality and homogeneity of variances were verified. Normality was assessed using the Shapiro–Wilk test, and variance homogeneity by the Levene test. Both tests confirmed that the data satisfied the required conditions, thus validating the use of ANOVA.

### 2.4. Adsorption Isotherm Studies

The adsorption behavior and mechanism of low-cost ceramic membranes were investigated using pre-prepared aqueous solutions of methylene blue (10–300 mg·L^−1^). Batch adsorption experiments were conducted at 25 °C with a contact time of 30 min, employing initial methylene blue concentrations of 10, 20, 50, 100, 150, 200, 250, and 300 mg·L^−1^. In each experiment, 2.25 cm^2^ (0.67 g) of ceramic membrane material was immersed in 60 mL of methylene blue solution and removed after reaching adsorption equilibrium. The solutions were subsequently filtered, and the residual dye concentrations were determined using a UV–Vis spectrophotometer (Pro Análise, Navegantes, Brazil).

The amount of methylene blue adsorbed (*q*) was calculated according to Equation (7) [36].

(7) $$q_{eq}=\frac{V}{m}\left(C_{0}-C_{f}\right)$$

where *C*_0_ (mg·L^−1^): Initial concentration of reactive dye solution, *C_f_* (mg·L^−1^): Final concentration remaining after the batch process concentrations, *q_eq_*: Quantity of adsorbed methylene blue (mg of dye/g of adsorbent), *V*: volume of dye solution (L), *m*: Mass of adsorbent (g).

## 3. Results and Discussion

### 3.1. Raw Materials Analysis

The particle size distribution (PSD) of the raw materials used in the membrane formulation is shown in Figure 1. Particle size plays a crucial role in ceramic processing, as it directly affects sintering behavior, porosity development, and the mechanical strength of the membranes.

**Figure 3:**
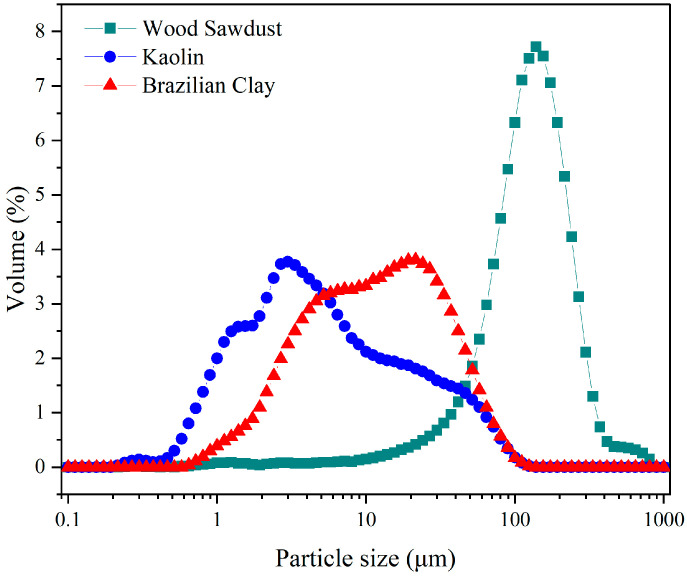
Particle size distribution of raw materials (Brazilian clay, kaolin, and wood sawdust).

The particle size distribution of Brazilian clay is illustrated in Figure 3. The data show that the particle diameter of Brazilian clay ranges from 0.644 μm to 100 μm, with average size of 18.59 μm. The particle size distribution of kaolin is illustrated in Figure 3. The data show that the particle diameter of kaolin ranges from 0.215 μm to 124.5 μm, with average size of 12.35 μm. The particle size distribution of wood sawdust is illustrated in Figure 3. The data show that the particle diameter of wood sawdust ranges from 0.24 μm to 803 μm, with average size of 152.7 μm [37,38,39].

Table 2 shows the chemical analysis results, which aim to identify the chemical constituents in the form of oxides.

The analysis clearly indicates that Brazilian clay primarily consists of silicon, aluminum, and iron oxides, due to the presence of clay minerals such as quartz, kaolinite, and smectite, respectively. The significant amount of Al_2_O_3_ in the sample (14.25%) is mainly aluminum combined in the structure with an exchangeable cation derived from the presence of clay minerals in samples [40,41,42]. The predominance of aluminum and silicon oxides suggests that the material is aluminosilicate [41]. Significant amounts of Fe_2_O_3_ were also found in Brazilian clay, indicating high iron content [41]. Calcium and magnesium oxides were found at concentrations below 2%, with these elements typically present as exchangeable cations [43,44].

Kaolin primarily consists of elements silica and alumina. Its composition features higher SiO_2_ percentage compared to Al_2_O_3_, resulting in an SiO_2_/Al_2_O_3_ ratio of nearly 1.

Wood sawdust is notably rich in CaO (61.11%), contains moderate amount of Fe_2_O_3_ (10.62%), and significant presence of other oxides such as K_2_O (8.19%) and SiO_2_ (5.53%).

**Figure 4:**
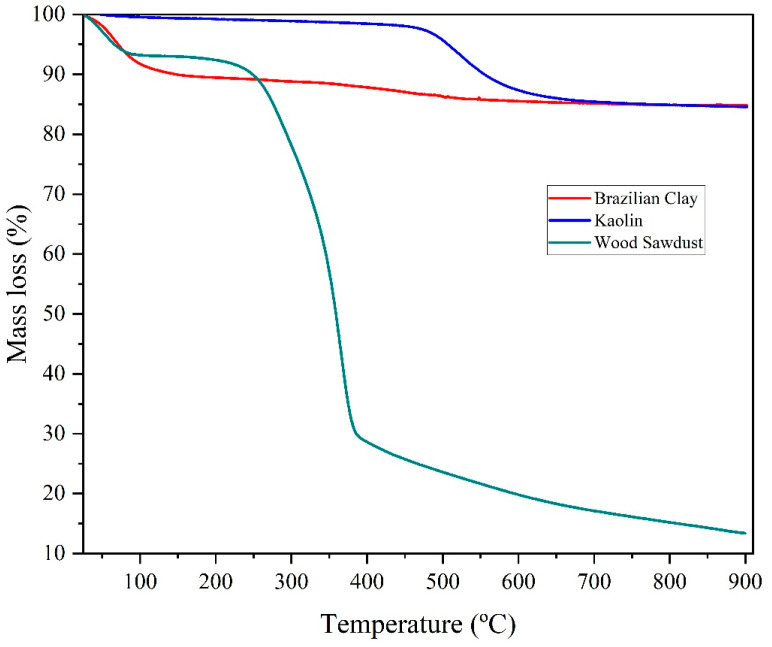
Thermogravimetric (TG) curves of Brazilian clay, kaolin, and wood sawdust.

Figure 4 shows the thermogravimetry (TG) results for Brazilian clay, kaolin, and wood sawdust.

Upon examining the thermogravimetric curve of Brazilian clay (Figure 4), three mass loss stages are evident. In the first stage, mass loss of 11.87% is observed within the temperature range of 124–178 °C, which is attributed to the loss of free water. The endothermic nature of this loss is reflected in the thermodifferential curve, occurring between 60 and 119 °C. Additionally, mass loss of 0.93% is observed between 124 and 178 °C, likely due to the loss of organic matter present in the clay.

In the second stage, further mass loss of approximately 5.26% is detected between 316 and 724 °C, corresponding to the loss of structural hydroxyl groups from clay minerals, with pronounced peak around 500 °C. The thermograms of Brazilian clay exhibit profile characteristic of clays containing minerals from the smectite group, showing total mass loss of 15.23% [40].

Upon examining the differential thermal analysis curves (Figure 4) for kaolin, minor mass loss of 0.96% is observed between 50–200 °C, attributed to the evaporation of free and adsorbed water. Subsequently, a significant peak, representing the most substantial mass loss in the range from 438 to 666 °C, is observed at 535 °C. The 12.06% mass loss is the result of the kaolinite dehydroxylation, marking the transformation of kaolin into metakaolinite in the range from 500 to 600 °C [45]. Additionally, an exothermic peak at approximately 993 °C signals the onset of mullite nucleation. The thermogravimetric analysis reveals total mass loss of 14.73%, accounting for the depletion of organic matter, water, and hydroxyl groups, corroborated by events outlined in the differential thermal analysis. The sample thermograms exhibit a characteristic curve profile, consistent with patterns reported in literature [46].

The TG curve represents the mass loss profile as a function of temperature [47]. As shown in Figure 3, the entire pyrolysis process can be divided into four stages. The first stage begins at 33 °C and ends at 200 °C, with mass loss of approximately 6.00%, primarily due to the removal of moisture and some light volatile compounds. The second stage occurs between 200 °C and 344 °C, with significant mass loss of 52.56%. The third stage occurs from 344 °C to 500 °C, with mass loss of 38.34%. The fourth stage begins at 500 °C and extends up to 800 °C, with mass loss of only about 1.00%, characterizing this phase as passive pyrolysis [48,49].

**Figure 5:**
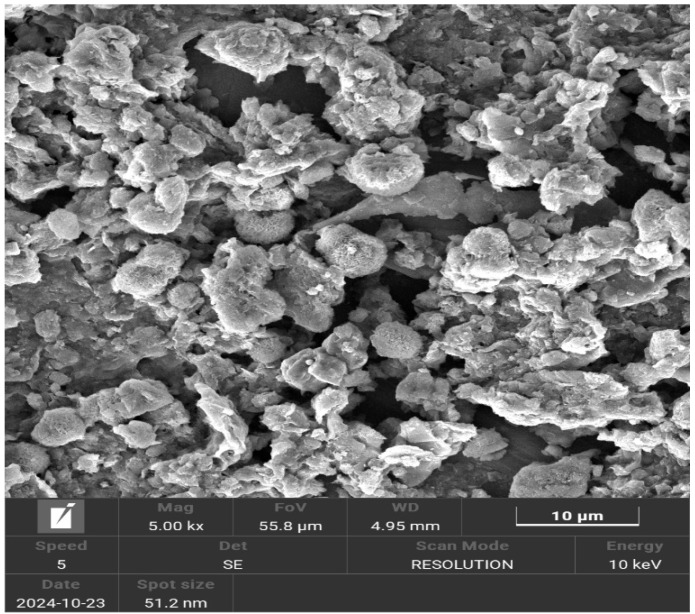
SEM image of Brazilian clay.

Figure 5 shows SEM images revealing the morphology of Brazilian clay. SEM micrographs reveal that the Brazilian clay consists of highly irregular lamellae of varying sizes, with some appearing partially curled [50].

**Figure 6:**
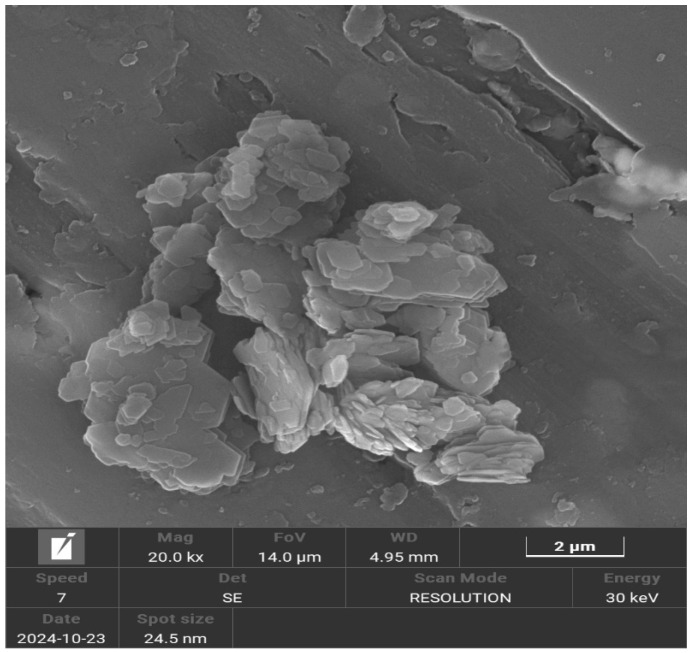
SEM image of kaolin.

Figure 6 shows the kaolin SEM-micrograph, which shows clusters of micrometric particles formed by the stacking of crystals that appear in a laminar shape with irregular, hexagon-like edges. These crystals exhibit a pseudo-hexagonal morphology, with particular emphasis on those that resemble booklets, a characteristic feature of kaolinite crystals [51].

**Figure 7:**
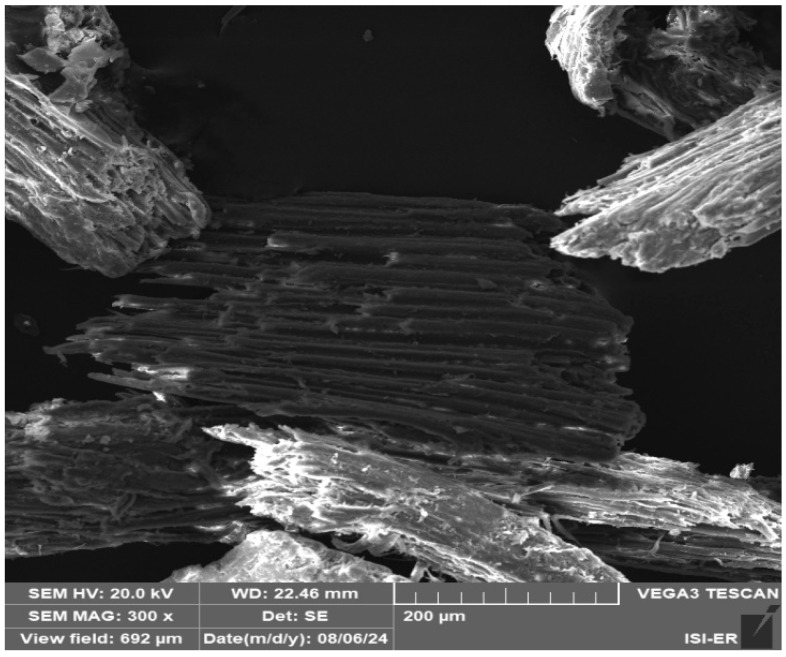
SEM image of wood sawdust.

The fiber morphology is displayed in Figure 7. Its surface resembles that of typical lignocellulosic fibers, featuring a longitudinal channel-like structure with visible surface defects.

**Figure 8:**
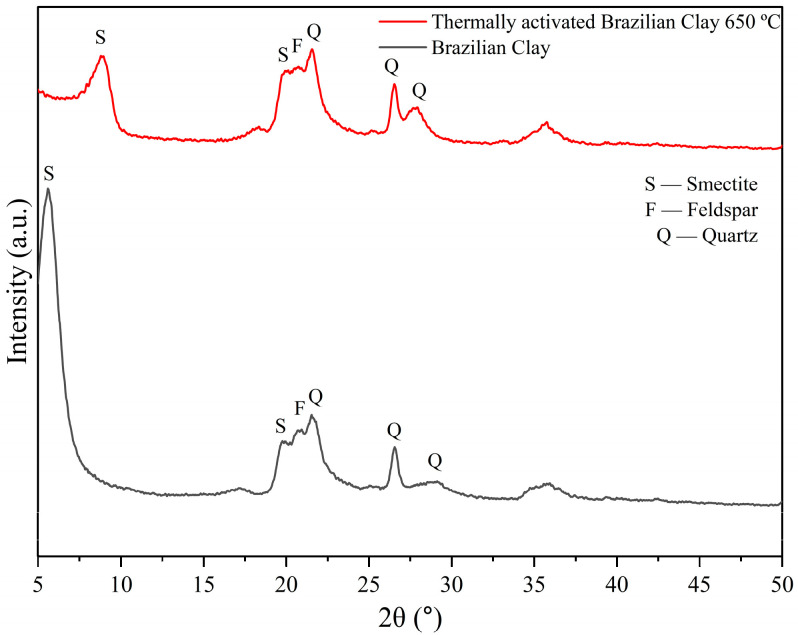
Diffractograms of Brazilian clay and thermally activated Brazilian clay at 650 °C.

Figure 8 displays the X-ray diffractograms of Brazilian clay and thermally activated Brazilian clay at 650 °C.

Analyzing the diffractograms of Brazilian clay presented in Figure 8, it is possible to observe the presence of peaks characteristic of smectite (JCPDFWIN ICDD 00-013-0135) and quartz (JCPDFWIN ICDD 01-078-2315), which are the main smectite clay mineral components. These peaks correspond to interplanar distances of d = 15.61 Å and 3.35 Å, respectively, which are characteristic of smectite clays [40,41,42]. Identification by X-ray diffraction revealed that the Brazilian clay consists of a mixture of clay minerals from the smectite group. Additionally, the presence of orthoclase (a type of feldspar) and α-quartz was observed. These findings are consistent with those reported in literature [44].

A common approach for physically modifying ceramic materials is thermal treatment. Heating at high temperatures alters the structure and composition of clay minerals [37]. During this process, all clay minerals undergo dehydration to varying degrees. In the higher temperature range, dehydration and dehydroxylation can occur simultaneously. These transformations, especially dehydration, can be manipulated for desired outcomes [42]. As shown in Figure 8, the smectite clay mineral peak intensity decreased after heating from 25 °C to 650 °C.

**Figure 9:**
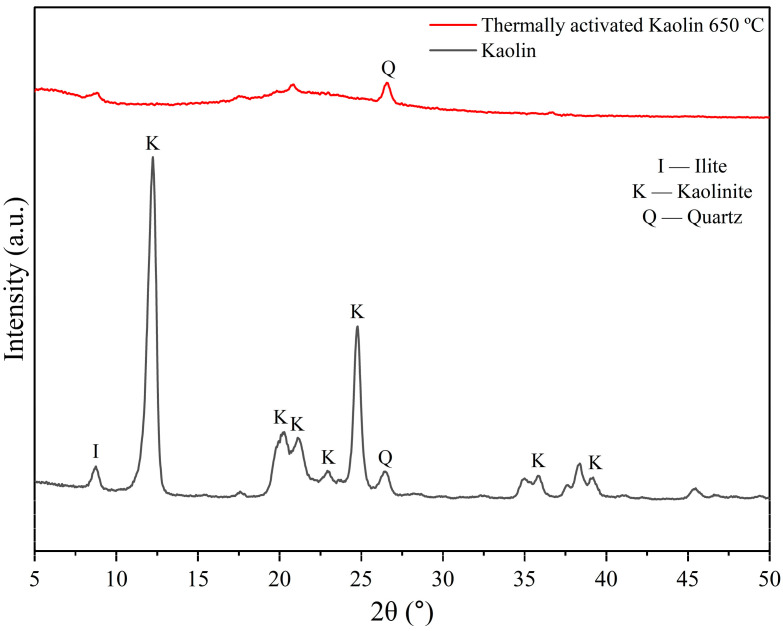
Diffractograms of kaolin and thermally activated kaolin at 650 °C.

Figure 9 shows the X-ray diffraction pattern from kaolin and thermally activated kaolin at 650 °C.

Analysis of the X-ray diffractograms reveals that kaolin (Figure 9) predominantly comprises the kaolinite mineral clay. This is evidenced by distinct reflections at 2θ = 12.41, 20.21 (multiple reflections), and 25.49°, with the first and last being notably intense and well-defined [52]. While other clay minerals like quartz (JCPDFWIN ICDD 87-2096) and illite (JCPDFWIN ICDD 02-0056) are present, their peaks are comparatively less intense than those of kaolinite. Minor traces of quartz are indicated by slight reflection at 2θ 26.5°, and illite is detected at 8.86° [52].

After heat treatment at 650 °C for 3 h, kaolin was converted into metakaolin (Figure 9) due to the collapse of its crystalline structure during the dehydroxylation process. Despite calcination, peaks corresponding to quartz and illite were still present, as the breakdown of these minerals requires temperatures above 650 °C. The pronounced curvature around 20° in 2θ indicated the presence of amorphous material. The transformation of kaolinite into metakaolin was confirmed by the absence of kaolinite diffraction peaks and the emergence of amorphous aluminosilicate [26]. Metakaolin, being amorphous, showed its strongest diffraction peaks due to quartz (SiO_2_), the crystalline phase in metakaolin.

**Figure 10:**
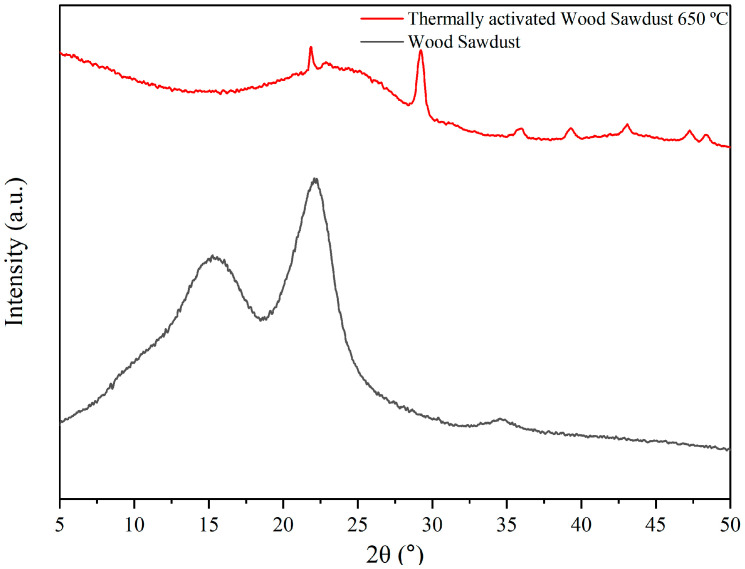
XRD patterns obtained for wood sawdust and thermally activated wood sawdust at 650 °C.

Figure 10 shows the X-ray diffraction analysis of wood sawdust and thermally activated wood sawdust at 650 °C.

The XRD patterns showed two prominent peaks at diffraction angles (2θ°) of 15.6° and 22.4°, corresponding to (101) and (002) lattice planes of cellulose I, respectively [53].

**Figure 11:**
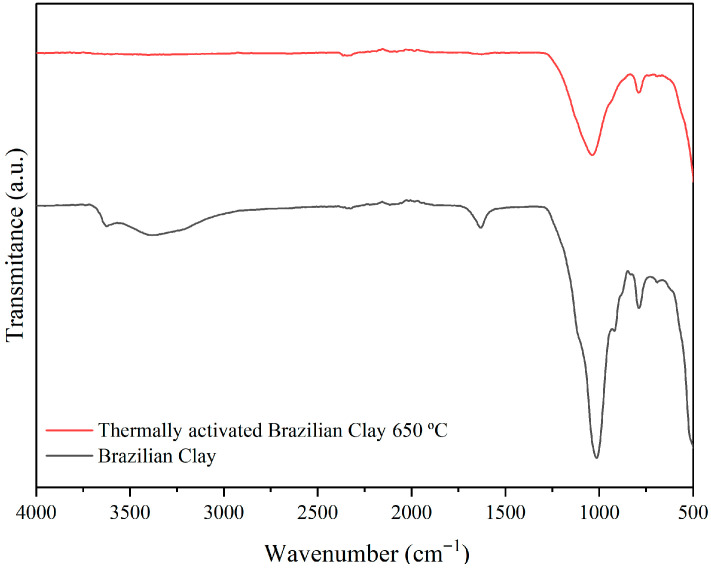
FTIR spectra of Brazilian clay and thermally activated Brazilian clay at 650 °C.

Figure 11 presents the FTIR spectra of Brazilian clay and thermally activated Brazilian clay in the range from 4000 to 500 cm^−1^ evaluated at room temperature.

The Brazilian clay shows bands in the 3630–3379 cm^−1^ and 1651 cm^−1^ regions, attributed to the stretching vibrations of structural hydroxyl groups and OH groups from adsorbed water. Bands in the 1019 cm^−1^ region are characteristic of Si–O–Si bonds, while those around 916 and 505 cm^−1^ correspond to the octahedral layers of aluminosilicate (Al–O–Si). These findings align with the previous literature [54]. Additionally, vibrations of the Si–O group (1019 cm^−1^) and octahedral layers (505 cm^−1^) can be observed in both samples.

Thermal treatment of Brazilian clay at 650 °C for 3 h is illustrated in Figure 11. The structural changes induced by smectite dehydration are evident in the FTIR spectra (Figure 11) of the tested sample. Heating the sample to 250 °C results in the removal of water molecules, which is reflected in the FTIR spectra of Brazilian clay by a decrease in the intensity of IR bands at 3630 cm^−1^ and 1635 cm^−1^, associated with –OH and –H–O–H groups, respectively.

**Figure 12:**
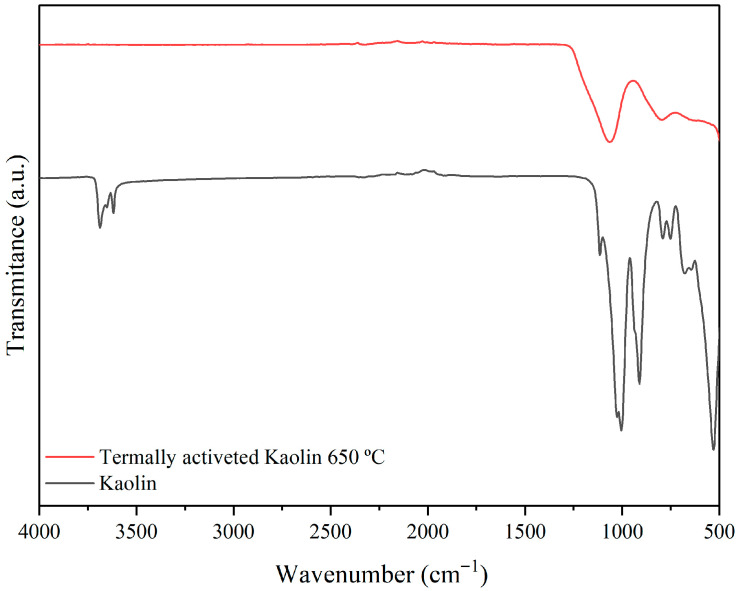
FTIR spectra of kaolin and thermally activated kaolin at 650 °C.

Figure 12 presents the FTIR spectra of kaolin and thermally activated kaolin in the range from 4000 to 500 cm^−1^ evaluated at room temperature.

The transformation of kaolin into metakaolin is evident from the IR spectra shown in Figure 12. The initial kaolin material exhibits well-defined IR bands in this range, corresponding to Si–O, Si–O–Al, and Al–OH vibrations. Upon conversion into metakaolin, these bands disappear, leaving a broad, intense, asymmetric band at 1112 cm^−1^ as the dominant feature. The loss of 1006 and 906 cm^−1^ bands is the result of the removal of Al–OH units, while alterations in Si–O stretching bands and the disappearance of Si–O–Al bands at 789 and 743 cm^−1^ reflect the distortion of tetrahedral and octahedral layers [55].

The thermal treatment of kaolin at 650 °C for 3 h transforms crystalline kaolin into amorphous metakaolin, accompanied by loss of internal water and dehydroxylation, as shown in Figure 12. In this process, the characteristic bands in the FTIR spectrum of kaolin disappear, and broad features emerge at approximately 1068 and 801 cm^−1^ (Figure 12).

**Figure 13:**
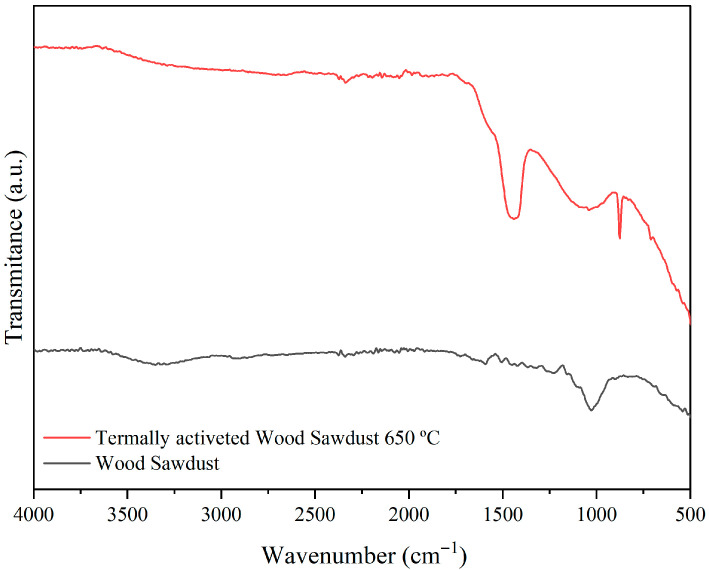
FTIR spectra of wood sawdust and thermally activated wood sawdust at 650 °C.

Figure 13 presents the FTIR spectra of wood sawdust and thermally activated wood sawdust in the range from 4000 to 500 cm^−1^ evaluated at room temperature.

The peak at 3325 cm^−1^ indicates the presence of hydroxyl groups (O-H), which may be associated with carboxylic acids, alcohols, and phenols found in cellulosic fibers, lignin, and pectins. The peak at 2916 cm^−1^ corresponds to CH_3_ stretching vibration. The peak at 1728 cm^−1^ is attributed to the stretching vibration of carboxylic acid and ester C-O bonds [19]. The peak at 1598 cm^−1^ represents the deprotonated carboxylate group (COO-). The peak at 1328 cm^−1^ suggests the stretching vibrations of pectin (-COOH) [20]. The peak at 1239 cm^−1^ indicates hemicellulose C-O stretching vibrations, highlighting the contribution of hemicellulose. Finally, peaks at 1024 and 535 cm^−1^ suggest presence of a halogen group (C-X) on the sawdust [21].

### 3.2. Low-Cost Ceramic Membranes Analysis

**Figure 14:**
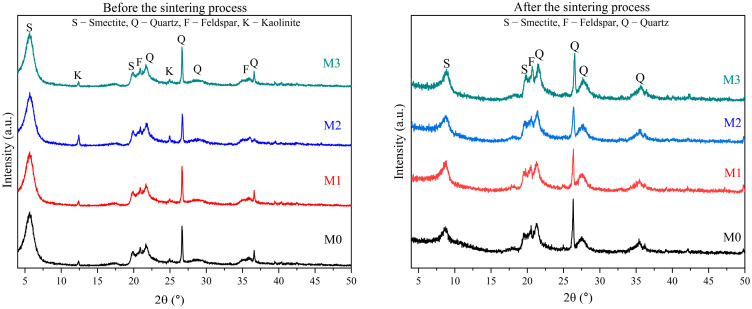
Diffractograms of M0, M1, M2, and M3 ceramic membranes before the sintering process and after the sintering process.

Figure 14 illustrates the X-ray diffractograms of low-cost ceramic membranes (M0, M1, M2, and M3) before and after the sintering process.

The X-ray diffractograms before the sintering process show characteristic peaks of smectite (S) and quartz (Q), the main components of the clay mineral smectite. Additionally, the presence of orthoclase (a type of feldspar) and α-quartz was observed. The presence of kaolinite was verified by characteristic reflections at 2θ = 12.41, 20.21 (multiple reflections), and 25.49°, with the latter partially overlapping the feldspar peak. Traces of quartz and illite were also observed, although their peaks were less pronounced compared to those of kaolinite, with the quartz peak coinciding with the peak of the clay mineral smectite. The lowest reflection at 2θ = 26.5° indicated the presence of traces of quartz, while 8.86° corresponded to illite. The same behavior is evident in formulations M1, M2, and M3, with the difference that sawdust peaks are present, observed at 2θ = 16° and 22.5° [56].

Before the sintering process, the XRD patterns of low-cost ceramic membranes (M0, M1, M2, and M3) exhibited no significant discrepancies, with distinct peaks corresponding to smectite clay mineral, kaolin, and sawdust, confirming their composition.

After analyzing the diffractograms of low-cost ceramic membranes after sintering shown in Figure 14, distinct peaks indicative of smectite (S) and quartz (Q), primary constituents of the smectite clay mineral, are evident. Additionally, the presence of orthoclase (a type of feldspar) and α-quartz is observed. Following the sintering process, all membranes exhibit elevated levels of smectite clay mineral, but quartz is also detected. These findings corroborate those reported in the literature [57].

Upon analyzing the XRD analysis of the M0 membrane, it becomes apparent that structural alterations occurred after the sintering process. A decrease in the peak associated with smectite clay is observed across all membranes, regardless of type. This observation highlights the impact of the sintering process, conducted under specific conditions, on the structural characteristics of Brazilian clay [57].

When comparing the feldspar peaks of membranes before sintering (Figure 14) to those after sintering (Figure 14), a minor disparity in intensity was observed in the primary peak positions. These results are consistent with prior research [58,59], providing additional validation and strengthening the robustness of findings.

The transformation of kaolin into metakaolin occurs due to the collapse of the kaolin crystalline structure as a result of the dehydroxylation process. This transformation is supported by the absence of kaolinite diffraction peaks and the appearance of amorphous aluminosilicate, as documented by the authors [60]. Metakaolin, being an amorphous material, demonstrates its main diffraction peaks due to the existence of quartz (SiO_2_), the crystalline phase in metakaolin.

Given this observation, it seems likely that similar phenomena occur with the other formulations, as raw materials remain consistent, except for the absence of wood sawdust in M0.

**Figure 15:**
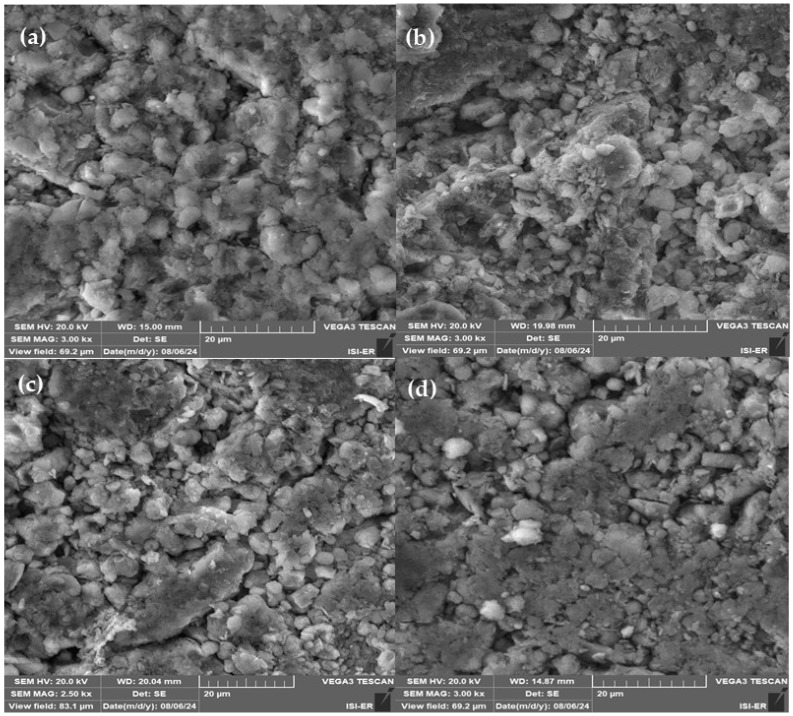
SEM surface images of the ceramic membranes: (**a**) M0, (**b**) M1, (**c**) M2, and (**d**) M3.

Figure 15 shows SEM surface images of low-cost ceramic membranes.

Electron microscopy analysis was performed to assess potential surface defects and verify pore formation on the membrane surface. SEM micrographs revealed surface heterogeneity across all manufactured membranes, along with pores of varying sizes. The images confirm the absence of cracks on membrane surfaces.

It is evident that a continuous porous structure was formed, with larger pores observed when wood was used as the pore-forming agent in membrane preparation (M1, M2, and M3) compared to those made without the addition of wood sawdust (M0). The incorporation of wood sawdust not only increases pore size but also enhances pore connectivity, due to the greater number of interconnected pores generated by the sawdust burnout, which is expected to improve permeability. This effect has been previously documented in the literature [18].

Porosity, average pore diameter, water uptake, mechanical strength, pure water flux, and hydrodynamic permeability values of low-cost membranes are summarized in Table 3.

The results shown in Table 3 indicate that incorporating wood sawdust waste into the membrane composition led to the following effects: (1) unchanged porosity unchanged; (2) increased average pore diameter; and (3) increased pure water flow rate.

The authors [23] developed low-cost membranes using sintering temperature of 850 °C, which is higher than the temperature used in this study. They reported porosity values ranging from 34.00% to 55.00%, which are consistent with results obtained in this work. However, the average pore diameter between 0.04 and 0.13 μm is smaller than values observed in this study.

The increase in pore size was correlated with the increase in water absorption (Table 3): from 30.21% (0% sawdust) to 33.22% (10% sawdust) for water absorption.

Porosity and pure water flow results of low-cost membranes reported by the authors [54] are similar to those found in this study. Notably, the pure water flow value is similar to results obtained for the M2 membrane.

Regarding mechanical resistance, progressive reduction in resistance was observed, where the M0 membrane, without wood sawdust, presented greater resistance, indicating that the addition of the porogenic agent reduced the value of this property. Despite the drop in resistance, all samples met the minimum requirement of 1.0 MPa established by the standard [61]. The mechanical resistance of a product depends on its microstructure and, mainly, on the distribution and size of defects present. Ceramic materials present a series of defects that can act as stress concentrators, determining the points where membrane fracture most easily begins [62].

In general, membranes with larger pores tend to exhibit higher hydrodynamic permeability, whereas denser and more compact membranes show lower L_p_ values [63]. It was observed that the permeability of the membranes increased with the amount of pore-forming agent added, with the densest sample, M0, showing the lowest L_p_ and the more open-pored sample, M3, exhibiting the highest values. Furthermore, hydrodynamic permeability was found to be closely related to both the average pore diameter and the overall porosity of the membrane [34].

The water contact angle was measured to assess the hydrophilicity of membranes. Membranes intended for liquid-phase microfiltration typically need to exhibit hydrophilic properties [64]. As expected, the M0, M1, M2, and M3 membranes displayed contact angles near 0°, indicating their hydrophilic nature, which is attributed to the presence of hydrophilic groups, particularly hydroxyl groups, enhancing their adsorption capacity. Water contact angles greater than 90° denote hydrophobicity, while angles below 90° indicate hydrophilicity. All membranes prepared in this study showed contact angles below 90°, confirming their hydrophilic nature and suitability for liquid-phase microfiltration.

### 3.3. Pure Water Permeate Flow

**Figure 16:**
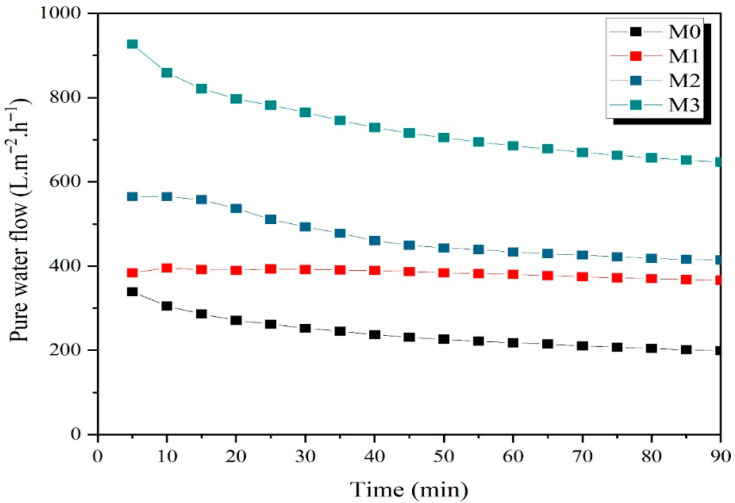
Pure water flow and permeate flow as a function of time for ceramic membranes.

The pure water flow as a function of time for low-cost ceramic membranes is shown in Figure 16. Experiments were carried out under the following conditions: pressure of 2.0 bar, temperature of 25 °C, and duration of 90 min.

In this study, the pure water flow values across ceramic membranes were determined to be 240.73 L/m^2^·h, 382.76 L/m^2^·h, 469.93 L/m^2^·h, and 732.89 L/m^2^·h for the M0, M1, M2, and M3 membranes, respectively.

The amount of wood sawdust used as a pore-forming agent was observed to impact the pure water flow through the membrane. A substantial increase in flow was observed with the addition of higher amounts of sawdust powder to the membrane formulation. It was also observed that, for permeate flow, membranes reached stability for around 60 min. This can be explained with reference to Table 3, which shows the increase in pore radius.

### 3.4. Treatment of Methylene Blue Wastewater by Ceramic Membranes

In this study, membranes were used to treat synthetic effluent containing blue methylene dye at a concentration of 100 mg·L^−1^. Experiments on membrane filtration were conducted at 25 °C, applying pressure of 2.0 bar. The four membranes, namely M0, M1, M2, and M3, which were sintered at 650 °C, were used for the experiments.

**Figure 17:**
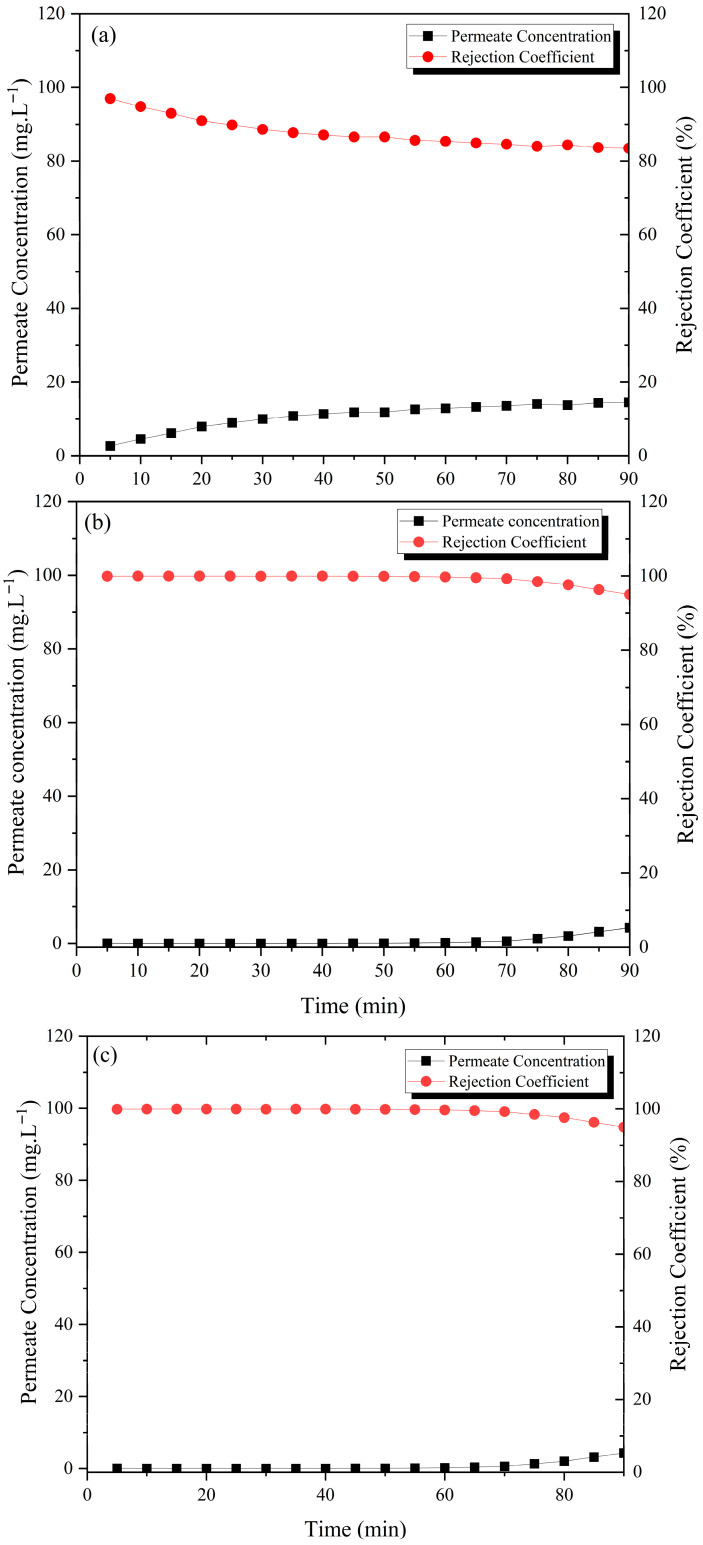

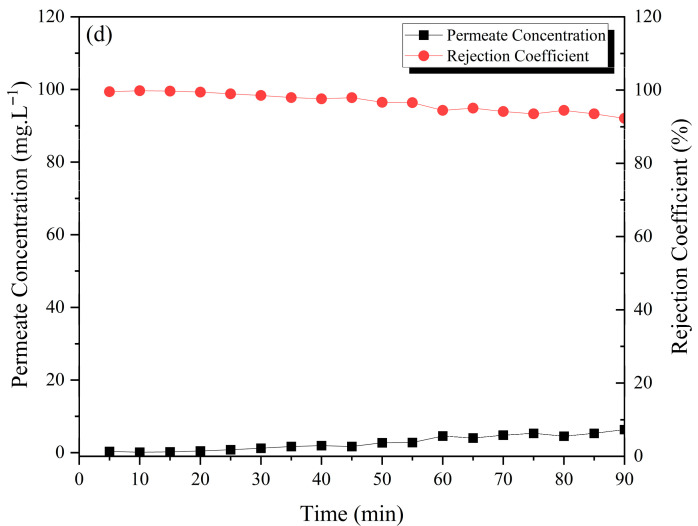
Dye rejection and permeate concentration as a function of time for ceramic membranes (**a**) M0, (**b**) M1, (**c**) M2, and (**d**) M3 [initial dye concentration = 100 mg·L^−1^, pressure = 2 bar, temperature = 25 °C, and pH = 6.8].

The permeate concentration e and the blue dye rejection coefficient over time for the filtration experiment with the M0, M1, M2, and M3 membranes are shown in Figure 17.

Using ceramic membranes for purifying water contaminated with methylene blue dye, initially at a theoretical concentration of 100 mg·L^−1^ and tested over 90 min, yielded the following results within the first five minutes: 0.660 mg·L^−1^ (a), 0.067 mg·L^−1^ (b), 0.065 mg·L^−1^ (c), and 0.352 mg·L^−1^ (d). These concentrations stabilized at 14.5 mg·L^−1^ (a), 6.56 mg·L^−1^ (b), 4.31 mg·L^−1^ (c), and 6.36 mg·L^−1^ (d). Thus, all membranes demonstrate significant dye removal efficiency. The membrane without the addition of sawdust exhibited the highest dye concentration in permeate value, with a decrease in dye concentration in permeate value observed as the sawdust percentage increased.

This trend was also observed for the rejection coefficient. Additionally, the process yielded average rejection values of 87.7% (a), 96.9% (b), 99.2% (c), and 96.7% (d).

Regarding the separation mechanism, it is evident that it is not sieving, as the average pore diameter of membranes ranges from 0.346 to 0.622 μm, whereas the molecular diameter of methylene blue is 0.008 μm.

Given that the membranes are composed of clay, metakaolin, and sawdust, and underwent sintering at temperature of 650 °C, it is plausible that all wood sawdust is removed according to literature data [65]. Therefore, all that remains is clay and metakaolin. Thus, it is conceivable to consider interaction between methylene blue and clay and metakaolin.

The surface chemistry of clay mineral particles plays critical roles in various agricultural, environmental, and technological processes. These processes are predominantly influenced by the reactivity of surface groups and the electrostatic interactions among particles, which arise from the distinctive charging properties of the clay mineral surface [66].

The pH value chosen for the tests was close to the isoelectric point (pHiep) of smectite clay, which was equal to 6 [67]. When the solution pH falls below pHiep, the clay carries a negative charge, whereas when the pH is higher than pHiep, it carries a positive charge.

The initial step occurs when dye is introduced into the clay suspension, leading to the adsorption of molecules onto the external surface of particles. This notably boosts local concentration, prompting the formation of MB aggregates such as trimers ((MB+)_3_) and dimers ((MB+)_2_). Over time, dye molecules may migrate to the interlamellar region, causing the disaggregation of aggregates and the return of protonated monomers due to increased acidity in this region. Consequently, adsorption proceeds via ion pairing mechanisms [68,69].

### 3.5. Data Analysis

When applying ANOVA (F = 20.26, *p* < 0.0001), a statistical difference was found among the membranes, as confirmed by the analysis. However, since four membranes were analyzed, it is necessary to identify which of them present specific statistical differences, as the F-test provides a global analysis. For a more detailed examination, the Tukey test was used. This test allowed the conclusion that the comparisons between M3 and the other three membranes (M2, M1, and M0) showed significant differences. This indicates that M3 has significantly different (and statistically superior) dye removal efficiency compared to the other membranes. The test also showed that there was no significant difference between M1 and M2.

### 3.6. Adsorption

A total of 0.6 g of the membrane was added to 60 mL of dye with a concentration of 100 mg·L^−1^. After adding the membranes (M0, M1, M2 and M3), the suspension (dye + membrane) was allowed to stir mechanically in an orbital shaker (150 rpm) for 30 min. The percentages of dye removal (R%) were 34.0%, 35.4%, 36.1% and 41.2%, respectively.

### 3.7. Adsorption Isotherm Studies

Different isotherm models, such as those of Langmuir and Freundlich, were employed to investigate the interactions between methylene blue and the ceramic membrane surface. The corresponding Equations (8) and (9) are presented below [70,71].

(8) $$q_{eq}=\frac{q_{max}K_{L}C_{e}}{1+K_{L}C_{e}}$$

where *q_eq_*: amount of reactive blue adsorbed per gram of adsorbent at equilibrium (mg·g^−1^); *q_max_*: maximum adsorption capacity (mg·g^−1^); *K_L_*: reactive blue/bauxite residue interaction constant (L·mg^−1^); *Ce*: dye concentration at equilibrium (mg·L^−1^).

(9) $$q_{eq}=K_{f}{\left(C_{e}\right)}^{\frac{1}{n}}$$

where *q_eq_*: amount of reactive blue adsorbed per gram of adsorbent at equilibrium (mg·g^−1^); *Ce*: dye concentration at equilibrium (mg·L^−1^); 1/*n*: constant related to surface heterogeneity; *K_f_*: Freundlich adsorption capacity constant (mg·L^−1^)·(L/g)^−1^.

**Figure 18:**
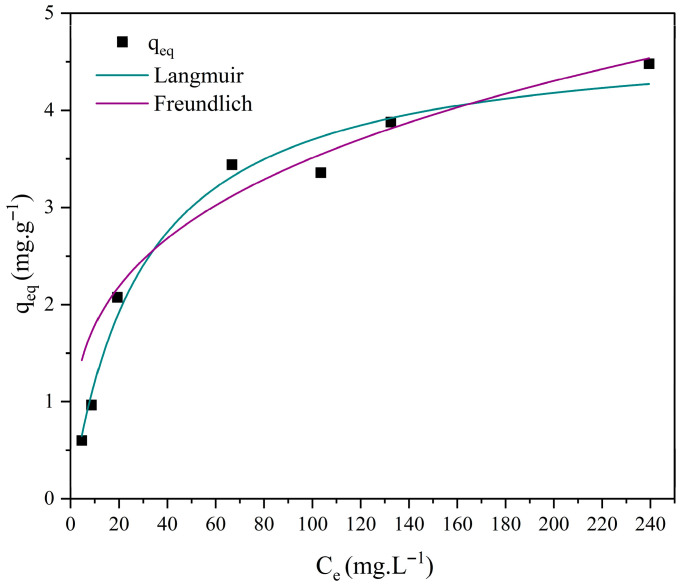
Adsorption isotherms of methylene blue using ceramic membrane M0 and nonlinear adjustments: Langmuir, Freundlich, and Temkin. [Batch system, T = 25 °C, initial concentration (C_0_) = 10 to 300 mg·L^−1^].

**Figure 19:**
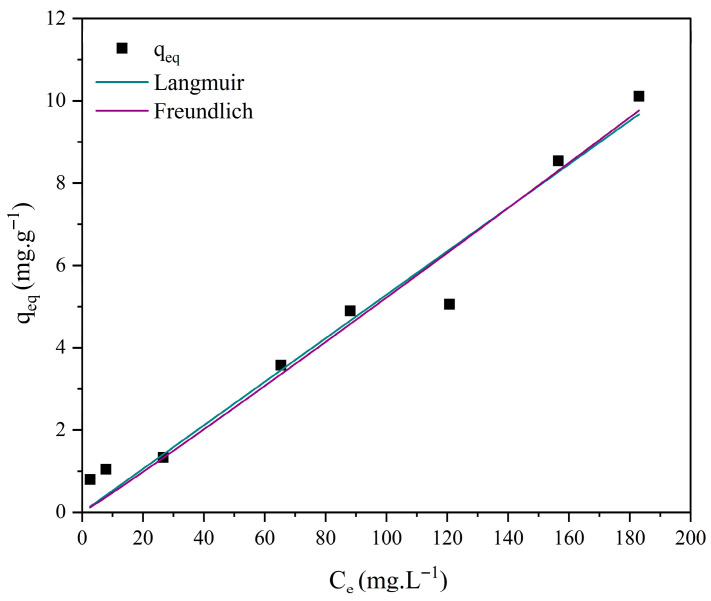
Adsorption isotherms of methylene blue using ceramic membrane M1 and nonlinear adjustments: Langmuir, Freundlich, and Temkin. [Batch system, T = 25 °C, initial concentration (C_0_) = 10 to 300 mg·L^−1^].

**Figure 20:**
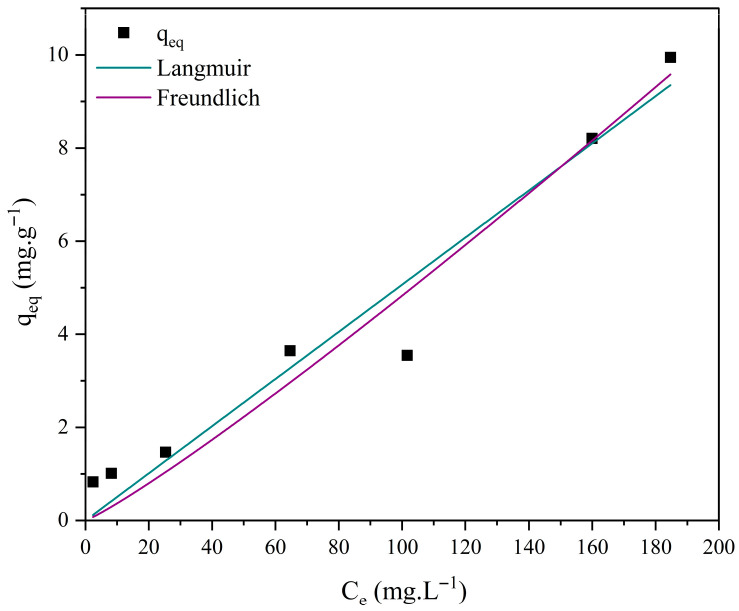
Adsorption isotherms of methylene blue using ceramic membrane M2 and nonlinear adjustments: Langmuir, Freundlich, and Temkin. [Batch system, T = 25 °C, initial concentration (C_0_) = 10 to 300 mg·L^−1^].

**Figure 21:**
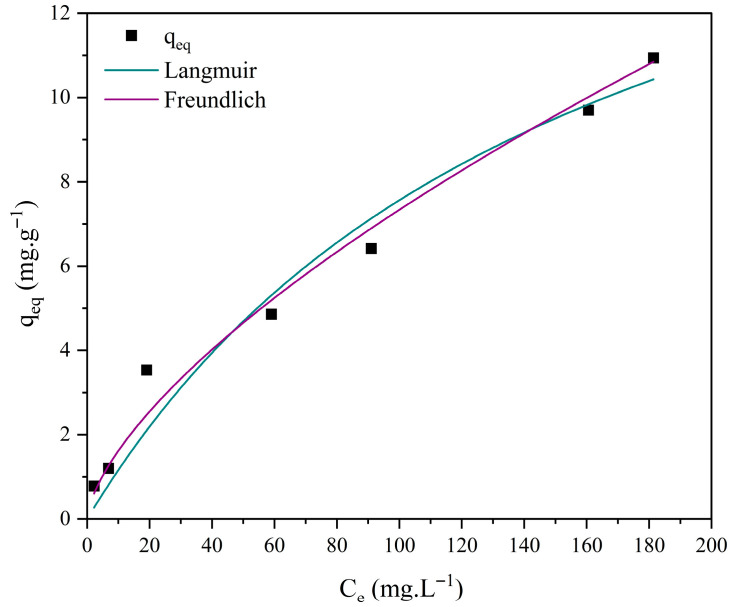
Adsorption isotherms of methylene blue using ceramic membrane M3 and nonlinear adjustments: Langmuir, Freundlich, and Temkin. [Batch system, T = 25 °C, initial concentration (C_0_) = 10 to 300 mg·L^−1^].

Figure 18, Figure 19, Figure 20 and Figure 21 display the experimental adsorption data along with the nonlinear fitting of the isotherm models.

The nonlinear adjustments of the Langmuir and Freundlich models to experimental data yielded parameters presented in Table 4.

Nonlinear fitting of the adsorption isotherms of methylene blue on ceramic membranes (M0, M1, M2, and M3) indicated that both the Langmuir and Freundlich models adequately represented the experimental data (Table 4). The R^2^ values confirmed that adsorption occurs on energetically homogeneous surfaces, predominantly through mono- and multilayer mechanisms.

**Figure 22:**
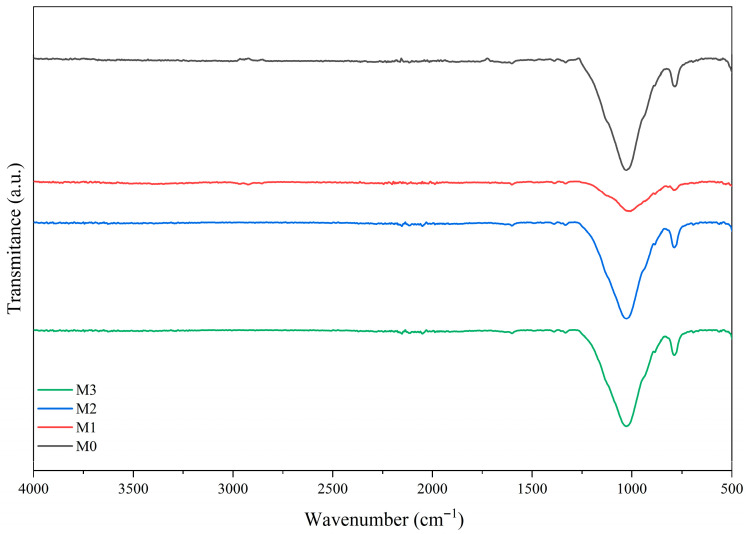
FTIR spectra of membranes (M0, M1, M2 and M3) after MB adsorption.

The functional groups in the membranes (M0, M1, M2 and M3) after MB adsorption were analyzed by FTIR, as shown in Figure 22.

After adsorption to methylene blue, the FTIR spectra of the membranes exhibited clear changes: the appearance or intensification of bands around 1500–1600 cm^−1^ (assigned to the aromatic vibrations of methylene blue) and an increase in the 1400–1470 cm^−1^ region, along with a reduction in the intensity of the O–H (~3400 cm^−1^) and H–O–H (~1600 cm^−1^) bands. Attenuation and a slight shift of the Si–O band (~1000–1100 cm^−1^) were also observed. These results suggest that dye retention involves electrostatic interactions and hydrogen bonding between methylene blue and the hydroxyl groups/sites of the clay, in addition to contributions from physical adsorption (surface coverage) and possible π–π interactions with the organic domains of the matrix.

### 3.8. Mechanism Analysis

Although both the Langmuir and Freundlich models adequately fit the experimental data, it is important to note that these models represent simplified mathematical approximations of the adsorption process. The fit to the Langmuir model suggests an initial monolayer adsorption on relatively homogeneous sites, while the Freundlich model reflects the heterogeneity of the ceramic surface and the possibility of multilayer adsorption at higher concentrations. However, given the complex pore structure and surface chemistry of ceramic membranes, the real adsorption mechanism is likely more intricate than either model individually predicts. Thus, the observed agreement with both models indicates a mixed mechanism, combining monolayer adsorption with multilayer deposition, but it does not fully capture the diversity of interactions between methylene blue molecules and the ceramic surface.

### 3.9. Role of Adsorption and Filtration Mechanisms in Dye Removal

Dye removal by batch adsorption over 30 min exhibited limited efficiency (35.0–41.2%), indicating a reduced availability of active adsorption sites. In contrast, continuous-flow filtration carried out for 90 min achieved removal efficiencies above 96.0%, with maximum values of 99.2%. Given that the average pore diameter of the membranes (0.346 μm, 0.450 μm, 0.495 μm, and 0.622 μm) is considerably larger than that of the methylene blue molecule (minimum diameter of 0.0008 μm), the separation process cannot be attributed to size exclusion [72]. Instead, the removal is governed by electrostatic and chemical interactions between the dye molecules and the mineral phases of the membranes (clay and metakaolin). The extended contact time, coupled with the convective transport generated under continuous flow, enhanced these interactions and led to higher removal efficiencies compared to batch adsorption. Nonetheless, the discrepancy in experimental durations (90 min for filtration versus 30 min for adsorption) should be taken into account when comparing the two processes.

It is important to acknowledge, however, that the study faced technical limitations in accurately identifying the types and distribution of elements within the modified membranes through EDX analysis. The overlapping signals of light elements, as well as the restricted spatial resolution of the technique, hindered a more precise characterization of elemental heterogeneity. These constraints should be considered when interpreting the adsorption mechanism, as they may limit the extent to which the structural and compositional changes in the membranes can be fully correlated with their adsorption performance.

### 3.10. Preliminary Cost Analysis

The manufacturing of ceramic membranes using commercially available materials such as silicon carbide, stainless steel, aluminum, zirconium oxide, and silicon nitride is highly expensive, usually ranging from USD 500 to USD 3000 per square meter. This strongly contrasts with the considerably lower cost range of USD 20 to USD 200 per square meter for polymeric membranes [68]. Moreover, the preparation process of ceramic membranes from these materials involves high sintering temperatures (1300–1700 °C), further raising production costs [55,73,74,75,76,77,78]. Despite the high initial costs, ceramic membranes offer advantages over their polymeric counterparts. These advantages include lower operating costs due to their extended useful life, ease of cleaning through high-temperature steam sterilization, and capability to restore initial permeability and water flow through reverse flushing and proper cleaning [79].

The inclusion of clay in ceramic membrane manufacturing has gained prominence due to its low production cost, as illustrated in Table 5. The cost-effectiveness of clay-based ceramic membranes emerges as a crucial factor driving their widespread adoption in membrane manufacturing.

The production cost of membranes outlined in this study is lower compared to that of other membranes [79,80]. As a result, these membranes can be manufactured at minimal cost, as they take advantage of membrane technology. The energy cost will naturally fluctuate depending on the country where membranes are manufactured.

Using clays as the primary material for ceramic membrane manufacturing is considered a viable solution.

The production costs of ceramic membranes shown in Table 5 reveal significant variability, reflecting the different costs involved in the fabrication process of clay-based ceramic membranes. A comprehensive assessment of production cost pricing should consider expenses such as raw materials, processing costs, maintenance, labor, and electricity [81].

## 4. Conclusions

Ceramic membranes obtained through dry uniaxial compaction were investigated in this study for the treatment of methylene blue dye-containing liquid effluents at the laboratory scale.

The properties of low-cost ceramic membranes were observed to be affected by the varying weight wood sawdust percentages.

It was observed that increasing the sawdust concentration in the form of powder did not result in increased porosity, but rather generated pores with larger radii [0.173–0.311]. During the flux of pure water using low-cost ceramic membranes, it became clear that the amount of sawdust used as a porogenic agent significantly alters the flux. The addition of 10% pore-forming agent promoted an increase from 240.63 L.m^−2^.h^−1^ (without additive) to 732.89 L.m^−2^.h^−1^, representing an increase of almost 3 times.

All membranes presented a totally hydrophilic character, with a contact angle of 0°, an estimated cost of 159 USD/m^2^, and a tendency for mechanical resistance to decrease with increasing pore-forming agent content (from 19.92 to 6.18 MPa).

The results suggested that integrating sawdust into the membrane composition significantly enhanced the methylene blue dye removal efficiency, with all membranes exceeding the average removal rate of 96%, highlighting the advantageous influence of sawdust.

Batch adsorption showed limited methylene blue removal (34.0–41.2%), while isotherm modeling indicated a mixed mechanism involving both mono- and multilayer adsorption. FTIR confirmed that electrostatic interactions, hydrogen bonding, and π–π interactions contributed to dye retention. In contrast, continuous-flow filtration achieved >96% removal, mainly due to enhanced contact time and convective transport. However, EDX limitations restricted a deeper understanding of the elemental distribution and its influence on adsorption performance.

The use of clays and sawdust in the development of membrane technology has attracted attention due to their low cost and low sintering temperature (650 °C). Furthermore, the use of sawdust also brings a relevant advance in waste management. Despite the favorable results related to the use of clay and sawdust as membrane precursors, sintering additives, membrane supports, and membrane filters in membrane technology, and their large-scale application, have not been thoroughly researched, implemented, and optimized.

In most cases, the energy used in sintering comprises most sintering expenses for ceramic membranes, thereby increasing manufacturing costs and restricting their potential applications. The incorporation of wood sawdust aims to lower manufacturing costs.

## Figures and Tables

**Table 1:** Proportion (%) of raw material in the composition of the ceramic masses used.

Formulations	Brazilian Clay (%)	Kaolin (%)	Pore-Forming Agent Wood Sawdust (%)
M0	95.0	5.0	0.0
M1	92.5	5.0	2.5
M2	90.0	5.0	5.0
M3	85.0	5.0	10.0

**Table 2:** Chemical composition of raw materials expressed in% of mass.

Sample	SiO_2_	Al_2_O_3_	MgO	CaO	Fe_2_O_3_	K_2_O	LOI
Brazilian clay	71.79	14.25	2.18	1.05	14.25	-	15.23
Kaolin	50.25	47.62	0.70	-	0.34	-	14.73
Wood sawdust	5.53	-	-	61.11	10.62	8.19	86.62

**Table 3:** Properties of ceramic membranes: pore diameter, porosity, water uptake, mechanical strength, water flux, and hydrodynamic permeability.

Membrane T_sintering_ (°C)	Pore Diameter (μm)	Porosity (%)	Water Uptake (%)	Mechanical Strength (MPa)	Pure Water Flux (L m^−2^ h^−1^)	Hydrodynamic Permeability (L m^−2^ h^−1^ bar^−1^)	Ref.
M0/650	0.346	42.96	30.21	19.92	240.73	120.36	This work
M1/650	0.450	40.85	29.02	15.25	382.76	190.80	This work
M2/650	0.495	41.48	31.47	10.68	469.93	235.80	This work
M3/650	0.622	40.99	33.22	6.18	732.89	366.48	This work
^1^ M/850	0.04–0.13	34.00–55.00	-	-	-	-	[23]
^2^ M1/650	-	42.51	-	-	546.00	-	[55]

^1^ M: Kaolin + feldspar + quartz + sawdust. ^2^ M1: Brasgel clay + corn starch.

**Table 4:** Values of parameters for each isotherm model used in this study.

Model	Parameters	M0	M1	M2	M3
Langmuir	$K_{L}\;(mg/g)$	0.033	7.533 × 10^−7^	1.788 × 10^−6^	0.006
	$q_{s\;}(mg/g)$	4.807	7.018 × 10^4^	2.833 × 10^4^	19.526
	R^2^	0.981	0.965	0.950	0.963
	χ^2^	0.049	0.491	0.793	0.693
Freundlich	$K_{F}\;(mg/g)$	0.908	0.044	0.028	0.357
	1/n	0.293	1.037	1.117	0.656
	n	3.406	0.964	0.895	1.523
	R^2^	0.939	0.966	0.952	0.994
	χ^2^	0.040	0.486	0.750	0.051

**Table 5:** Cost of different clay-based low-cost ceramic membranes.

Ceramic Membrane	Membrane Type	Cost of the Membrane (USD/m^2^)	Refs.
Fly ash-based low-cost ceramic	Tubular	250	[79]
Fly ash-based ceramic	Disc	225	[80]
Low-cost ceramic membrane	Disc	159	This study

## Data Availability

The original contributions presented in this study are included in the article. Further inquiries can be directed to the corresponding author.
